# Chorioamnionitis Precipitates Perinatal Alterations of Heme-Oxygenase-1 (HO-1) Homeostasis in the Developing Rat Brain

**DOI:** 10.3390/ijms22115773

**Published:** 2021-05-28

**Authors:** Maide Ozen, Yuma Kitase, Vikram Vasan, Christopher Burkhardt, Sindhu Ramachandra, Shenandoah Robinson, Lauren L. Jantzie

**Affiliations:** 1 Department of Pediatrics, Neonatal-Perinatal Medicine, Johns Hopkins University School of Medicine, Baltimore, MD 21205, USA; ykitase1@jhmi.edu (Y.K.); vikramvasan7@gmail.com (V.V.); cburkh11@gmail.com (C.B.); sindhuramachandra@usf.edu (S.R.); srobin81@jhmi.edu (S.R.); LJantzie@jhmi.edu (L.L.J.); 2 Department of Neurosurgery, Johns Hopkins University School of Medicine, Baltimore, MD 21287, USA; 3 Kennedy Krieger Institute, Baltimore, MD 21205, USA; 4 Department of Neurology, Johns Hopkins University School of Medicine, Baltimore, MD 21205, USA

**Keywords:** HO-1, TfR1, neuroinflammation, peripheral immune activation, perinatal brain injury, neurodevelopment, neural-immune

## Abstract

Chorioamnionitis (CHORIO), placental insufficiency, and preterm birth are well-known antecedents of perinatal brain injury (PBI). Heme-oxygenase-1 (HO-1) is an important inducible enzyme in oxidative and inflammatory conditions. In the brain, HO-1 and the iron regulatory receptor, transferrin receptor-1 (TfR1), are known to be involved in iron homeostasis, oxidative stress, and cellular adaptive mechanisms. However, the role of HO pathway in the pathophysiology of PBI has not been previously studied. In this study, we set out to define the ontogeny of the HO pathway in the brain and determine if CHORIO changed its normal developmental regulation. We also aimed to determine the role of HO-1/TfR1 in CHORIO-induced neuroinflammation and peripheral inflammation in a clinically relevant rat model of PBI. We show that HO-1, HO-2, and TfR1 expression are developmentally regulated in the brain during the perinatal period. CHORIO elevates HO-1 and TfR1 mRNA expression in utero and in the early postnatal period and results in sustained increase in HO-1/TfR1 ratios in the brain. This is associated with neuroinflammatory and peripheral immune phenotype supported by a significant increase in brain mononuclear cells and peripheral blood double negative T cells suggesting a role of HO-1/TfR1 pathway dysregulation in CHORIO-induced neuroinflammation.

## 1. Introduction

Perinatal infection and inflammation have central roles in adverse neurodevelopment. In human very low birth weight (VLBW) neonates, inflammation and hypoxia-ischemia result in a cumulative increase in the risk of cerebral palsy (CP) [1]. Preterm neonates are at increased risk for motor and cognitive impairment of which the odds are exacerbated in the presence of exposure to chorioamnionitis [2,3]. The concept of intra-uterine sterile inflammation has been emphasized by a number of researchers in the etiopathogenesis of preterm labor and premature birth [4,5]. Acute chorioamnionitis, the inflammation of the chorioamnion, can extend to the villi (acute villitis), to umbilical cord (funisitis) and result in a systemic fetal inflammatory response syndrome (FIRS) [4]. Fetal inflammatory response syndrome type 1, with associated increases in amniotic fluid and fetal serum IL-6, is associated with acute chorioamnionitis [5]. FIRS is an independent predictor of neonatal morbidity, and significantly increases the risk of periventricular leukomalacia (PVL) and cerebral palsy (CP) [6]. Therefore, it is imperative that we define novel molecular mechanisms involved in CHORIO-induced preterm brain injury, which may aid in developing innovative diagnostic and therapeutic interventions.

Heme-oxygenase, an evolutionary conserved pathway, has multitude of crucial functions in the body [7]. Heme-oxygenase is an enzyme with two functional isozymes [8]. HO-1, the inducible isoform is present in the central nervous system (CNS) and predominantly expressed by astrocytes, oligodendrocyte precursor cells, microglia/macrophages and endothelial cells [9]. HO-2, is constitutively expressed in all tissues [8]. A third form, HO-3, has also been described in the literature [8]. HO-1 homeostasis is important for establishment of a healthy fetoplacental interface [10,11]. HO-1 also has essential roles in preventing apoptosis and oxidative stress, providing cytoprotection, maintaining mitochondrial homeostasis and regulating overall immune system balance [10,12,13]. In the brain, HO-1 and the iron regulatory receptor, transferrin receptor-1 (TfR1), are known to be involved in cellular adaptive mechanisms. Specifically, a sustained, prolonged, uncontrolled increase in HO-1 is detrimental such that it can promote a pro-inflammatory pre-disposition by inducing iron-dependent lipid peroxidation, cytotoxicity and cell death [14,15,16,17,18]. TfR1 was recently identified as a specific ferroptosis marker [19]. Additionally, like HO-1, TfR1 is also important in the immune system homeostasis. While HO-1 regulates both the innate and adaptive arms of the immune system, TfR1 is important in adaptive immune system [20]. Dysregulation of HO-1 homeostasis is linked to many pregnancy complications, neonatal and adult diseases, including neurodegenerative and neuroinflammatory diseases of the CNS, such as Parkinson’s, Alzheimer’s, and multiple sclerosis [7,10,13,21,22].

HO-1 is developmentally regulated in the rat brain at normal baseline conditions, in the absence of infection or inflammation [23]. It is important to note that maintaining optimal HO-1 homeostasis and the regional expression patterns of HO-1 within the brain remains important across the life span for rodents and humans. Previously, we have shown that transient placental insufficiency concomitant with intra-amniotic injection of the bacterial endotoxin lipopolysaccharide (LPS) at embryonic day (E) 18 results in histologic chorioamnionitis and an initial type 1 FIRS [24]. These changes in placental pathology and FIRS occurred along with a sustained systemic inflammatory response, brain injury, MRI abnormalities, and functional impairment [24,25,26,27,28]. Importantly, brain injury in this model accurately outlines the histopathologic features, motor and functional consequences of cerebral palsy in extreme prematurity [26,27]. Hence, this represents a clinically relevant, validated model of CP to study the HO pathway alterations in CHORIO. However, the gap in our knowledge remains for the exact mechanistic underpinnings of CP associated with chorioamnionitis in vulnerable, extremely premature born neonates. In this study, our objectives were to first, define the ontogeny of the HO pathway in the rat brain and to test whether chorioamnionitis altered its normal developmental regulation. Second, we aimed to determine CHORIO-induced changes in neuroinflammation and peripheral inflammation. We hypothesized that CHORIO would alter the normal HO pathway homeostasis in the developing rat brain and specifically would result in a sustained upregulation of HO-1 in neonatal period, which would be associated with sustained postnatal pro-inflammatory programming of the CNS and peripheral immune system. To our knowledge, this study reports for the first time a loss of the normal developmental regulation of HO-1/TfR1 in the developing CNS after CHORIO using an established and translational model of CP.

## 2. Results

### 2.1. Placenta HO-1, HO-2, and TfR1 Expression

**Figure 1:**
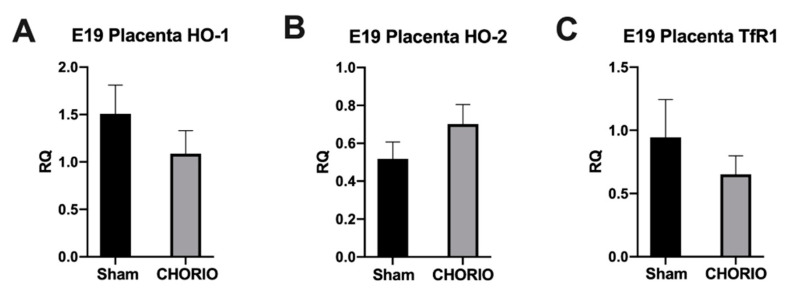
Placenta gene expressions for HO-1, HO-2, and TfR1 24 h after CHORIO, at E19, 24 h after CHORIO, at E19, no significant differences in placental gene expressions of HO-1 ((**A**) *n* = 4 for CHORIO, *n* = 3 for sham), HO-2 ((**B**) *n* = 4 for CHORIO, *n* = 3 for sham) or TfR1 ((**C**) *n* = 3 for CHORIO, *n* = 3 for sham) was detected compared to sham. *p* > 0.05, data normally distributed, Welch’s *t*-test, 2-tailed, mean ± SEM.

The diagnosis of acute chorioamnionitis in placenta pathology of preterm neonates who are born less than 28 weeks of gestational age is exceptionally high [4]. Intra-amniotic infection/inflammation can progress to choriodecidual membranes and subsequently FIRS [4]. It was previously shown that the risk of preterm delivery in fetuses with FIRS within next 48 h was higher when compared with fetuses that did not have FIRS [6]. Thus, we first studied placental gene expression. Twenty-four hours after induction of CHORIO and in sham controls at E19, we measured HO-1, HO-2, and TfR1 mRNA expression in placenta. We did not detect a significant difference between CHORIO placenta HO-1 mRNA (1.087 ± 0.2422, *n* = 4), Figure 1A, HO-2 mRNA (0.7023 ± 0.1023, *n* = 4), Figure 1B, or TfR1 mRNA (0.6523 ± 0.1459, *n* = 3), Figure 1C, expression at E19 compared to sham HO-1, HO-2, and TfR1 (1.508 ± 0.3038, 0.5178 ± 0.08944, 0.9440 ± 0.3, *n* = 3), respectively.

#### 2.1.1. Normal Developmental Regulation of HO-1 in Rat Brain

**Figure 2:**
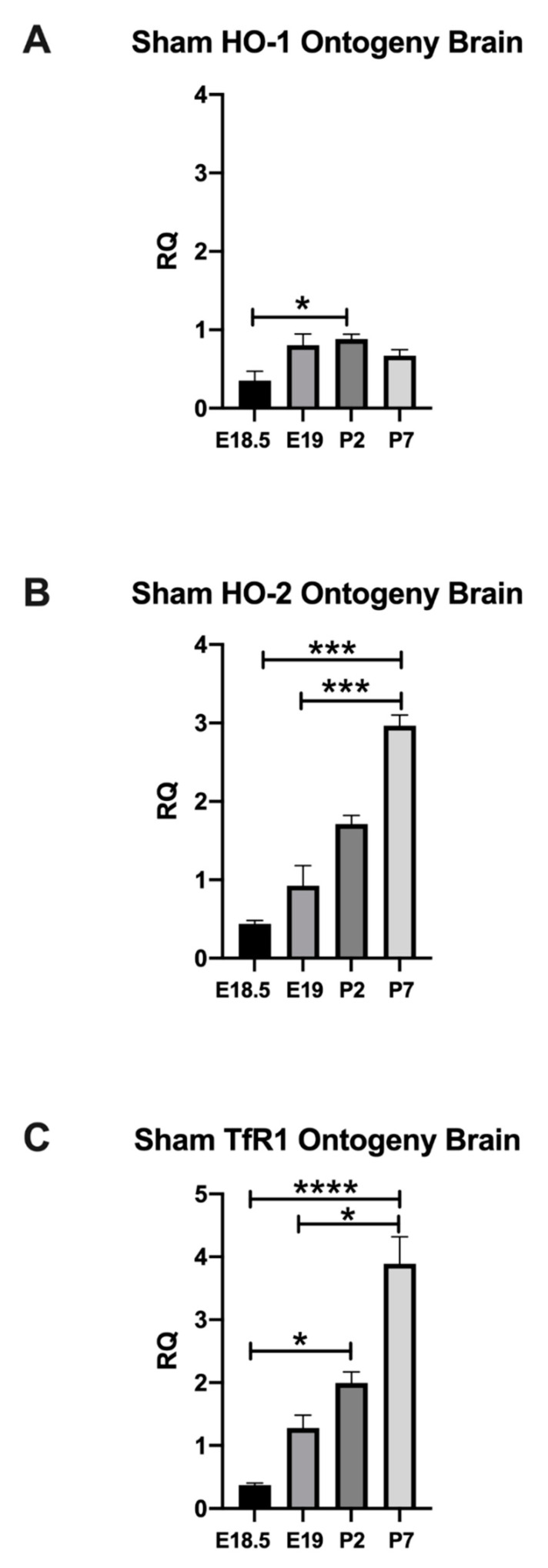
Ontogeny of HO-1, HO-2, and TfR1 in Sham Brains. Normal developmental regulation of HO-1 ((**A**) *n* = 3, 5, 10, 7, P2 vs. E18.5, * *p* = 0.0394), HO-2 ((**B**), *n* = 6, 8, 7, 7, P7 vs. E18.5, *** *p* = 0.0006, P7 vs. E19, *** *p* = 0.0009), and TfR1 ((**C**), *n* = 7, 8, 6, 7, P2 vs. E18.5, * *p* = 0.0169, P7 vs. E18.5, **** *p* < 0.0001, P7 vs. E19, * *p* = 0.0275) is displayed at E18.5, E19, P2, and P7 respective postnatal ages. *p* significant when <0.05, Kruskal–Wallis test, mean ± SEM.

As HO-1 is an inducible stress enzyme, we then studied HO-1 mRNA expression in sham cortex to establish baseline developmental values prior to assessing changes with in utero injury. In sham cortex, HO-1 mRNA was significantly increased at P2 (0.8811 ± 0.06468) compared with E18.5 (0.3513 ± 0.1218). Specifically, compared to E18.5, HO-1 mRNA was increased by 150.8% at P2 (*p* = 0.0394), Figure 2A.

#### 2.1.2. Normal Developmental Regulation of HO-2 in Rat Brain

HO-2 is the isozyme that is constitutively expressed. Therefore, we established the developmental regulation of the constitutive HO-2 in sham cortex, next. In sham cortex, HO-2 mRNA at P7 (2.964 ± 0.1378) was significantly higher when compared with E18.5 (0.4393 ± 0.04362) and E19 (0.9256 ± 0.2564), a 574.71% and 220.225%, respectively (*p* = 0.0006 and 0.0009), Figure 2B.

#### 2.1.3. Normal Developmental Regulation of TfR1 in Rat Brain

After examining the developmental regulation of HO-1 and HO-2 mRNA, we studied the changes in expression of TfR1 mRNA, the cellular receptor for heme [29]. In sham cortex, TfR1 mRNA was significantly upregulated at P2 (1.995 ± 0.1746) and at P7 (3.890 ± 0.4291) when compared with E18.5 (0.3743 ± 0.03115), a 432.995% and 939.273% rise respectively, *p* = 0.0169 and <0.0001. Additionally, we detected a 204.144% rise at P7 (3.890 ± 0.4291) when compared with E19 (1.279 ± 0.2047), *p* = 0.0275, Figure 2C.

These data demonstrate that HO-1, HO-2, and TfR1 are developmentally regulated in the fetal and neonatal sham brains. They significantly increase through the fetal (E18.5) and early neonatal periods. Indeed, the time period between E18 to P7 highlights a critical CNS development period in rodents, which would be human equivalent of a time span between 24 weeks to term equivalent age [26]. Hence, developing brain is susceptible to insults during this critical maturation window and peak time of preterm birth. Cellularly, this rapid period of development corresponds to the emergence of pre-oligodendrocytes and neuronal migration to name a few indicating the importance of maintaining cellular homeostasis during this critical developmental window. Since HO-1, HO-2, and TfR1 are developmentally regulated this may suggest that they are important in maturation, migration, and/or development of these cellular subsets [26].

### 2.2. CHORIO Altered the Ontogeny of HO-1, HO-2, and TfR1 in Rat Brain

After establishing the changes in the HO pathway in the third trimester equivalent developmental window, we then assessed maturational alterations in cerebral HO-1, HO-2, and TfR1 mRNA expression in CHORIO. For HO-1 mRNA expression, we discovered a significant injury effect in CHORIO compared with sham (*p* = 0.0092), by two-way ANOVA, Supplementary Figure S1A. Whereas, for HO-2, Figure S1B and TfR1, Figure S1C mRNA expression, we detected a significant effect of time.

**Figure 3:**
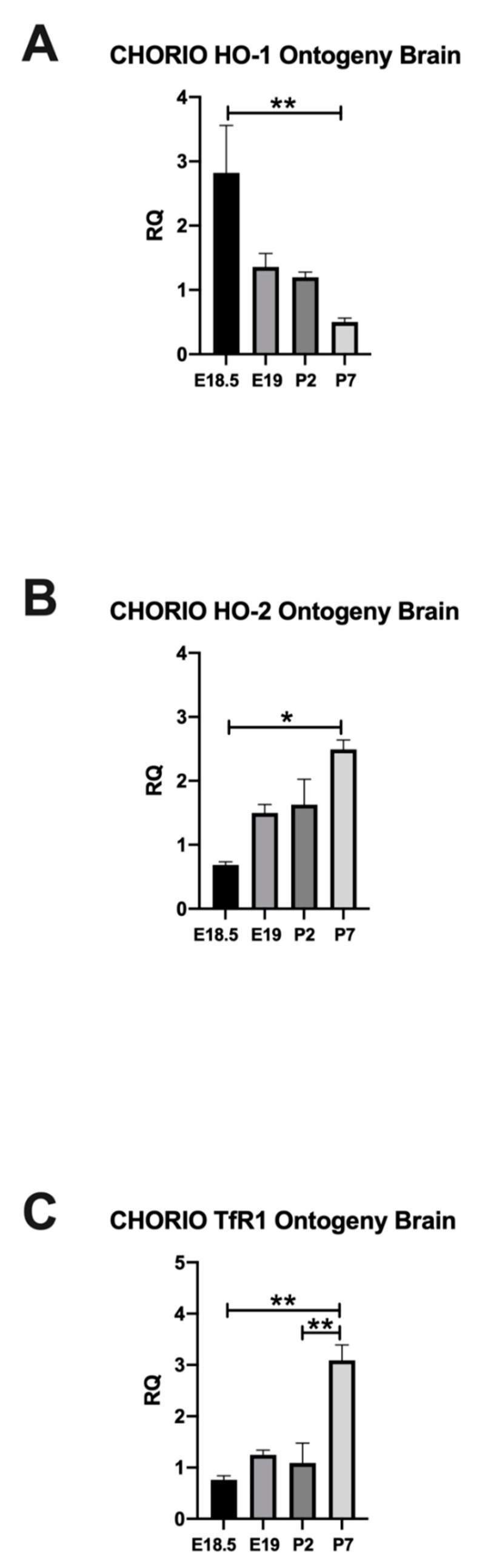
Ontogeny of HO-1, HO-2, and TfR1 in CHORIO. Effect of CHORIO on the developmental regulation of HO-1 ((**A**), *n* = 8, 4, 3, 7, E18.5 vs. P7, ** *p* = 0.0028), HO-2 ((**B**), *n* = 6, 3, 5, 7, P7 vs. E18.5, * *p* = 0.0011) and TfR1 ((**C**), *n* = 7, 6, 6, 7, P7 vs. E18.5, ** *p* = 0.0022, P7 vs. P2, ** *p* = 0.005) is displayed at E18.5, E19, P2, and P7 respective postnatal ages. *p* significant when <0.05, Kruskal–Wallis test, mean ± SEM.

CHORIO significantly impacted the normal developmental expression of HO-1 (2.822 ± 0.735) in the brain for E18.5 compared to P7, Figure 3A. However, the developmental regulation of the constitutive HO-2 in the brain after CHORIO was preserved at E18.5, E19, and P2 (0.6867 ± 0.04853, 1.498 ± 0.1343, 1.628 ± 0.3957), Figure 3B. Similar to sham brains, HO-2 in CHORIO brains, reached highest value at P7 compared to E18.5 (*p* = 0.0011), Figure 3B. Likewise, TfR1 in CHORIO brains were highest at P7 among studied timepoints (*p* = 0.0022 E18.5 vs. P7 and 0.005 P2 vs. P7), Figure 3C, injury changed HO-1 but not HO-2 or TfR1. Thus, we showed an early alteration in the mRNA expression of this essential homeostatic enzyme in the developing brain and at the peak time for preterm birth.

#### 2.2.1. HO-1 Expression in Rat CHORIO Brain

**Figure 4:**
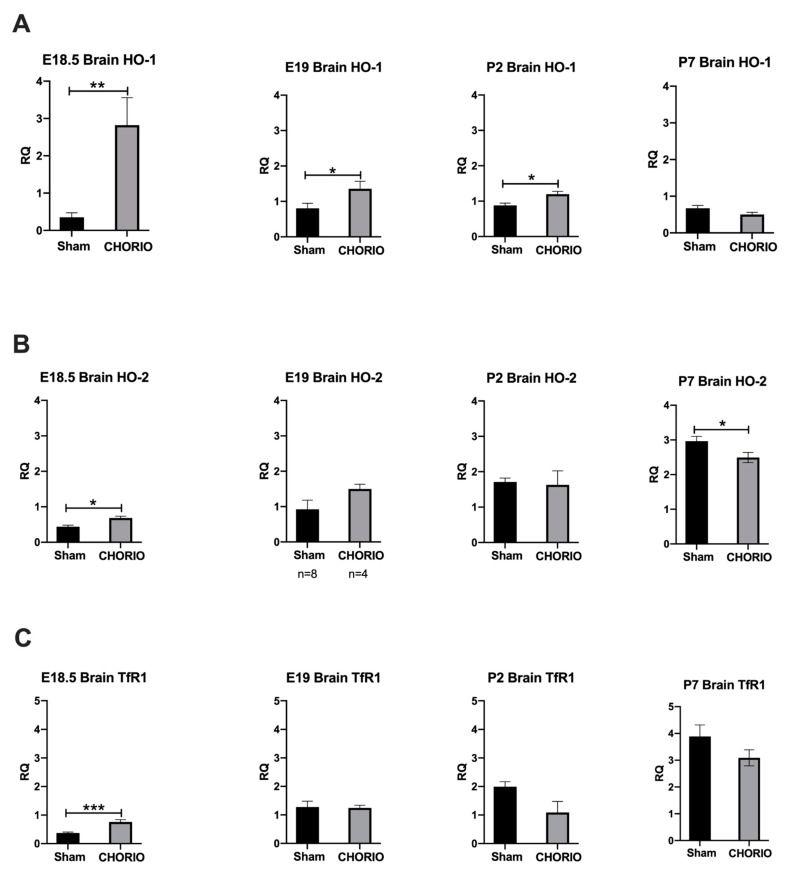
CHORIO alters HO-1, HO-2, and TfR1 expressions in brain compared to Sham. At E18.5, CHORIO upregulated HO-1, ** *p* = 0.0061 (**A**), HO-2, * *p* = 0.0152 (**B**), and TfR1, *** *p* = 0.0006 (**C**) in brains compared with sham. At E19 and P2, CHORIO upregulated HO-1, * *p* = 0.0317 and * 0.0490, respectively (**A**) in brains compared with sham. At P7, CHORIO downregulated constitutional HO-2 expression, * *p* = 0.0175 (**B**) significantly compared with sham. *p* significant when < 0.05, Kruskal–Wallis test, mean ± SEM.

HO-1 expression in CHORIO brains at E18.5 was significantly higher, 703.302% (2.822 ± 0.735, *n* = 8) compared with sham controls (0.3513 ± 0.1218, *n* = 3), *p* = 0.0061. Likewise, at E19 (24 h after), CHORIO brains continued to have significantly increased HO-1 expression, 68.6972% (1.357 ± 0.2117) compared with GA-matched sham brains (0.8044 ± 0.1437), *p* = 0.0317. Significant HO-1 upregulation in CHORIO brains continued at P2, 36.0799% (7-days after the initial insult, 1.199 ± 0.07959) compared with postnatal age matched-shams (0.8811 ± 0.06468), *p* = 0.0490. HO-1 in CHORIO brains were similar to sham brains 12-days after initial insult at P7 at term equivalent age in humans, *p* > 0.05, Figure 4A.

#### 2.2.2. HO-2 Expression in Rat CHORIO Brain

Six hours after induction of CHORIO, at E18.5, HO-2 was significantly increased, 56.3169% (0.6867 ± 0.04853) compared with sham brains (0.4393 ± 0.04362, *p* = 0.0152). Whereas, HO-2 expression at E19 and P2 brains were similar in CHORIO and sham when compared at their respective gestational and post-natal ages. However, 12-days after initial insult at P7, constitutive HO-2 in CHORIO brains were significantly lower (2.493 ± 0.1474) with a 14.74008% decrease compared with sham (2.964 ± 0.1378, *p* = 0.0175), Figure 4B.

### 2.3. TfR1 Expression in Rat CHORIO Brain

At E18.5, 6 h after induction of CHORIO, brain TfR1 was significantly higher, 103.4999% increase (0.7617 ± 0.07796) compared with GA-matched sham brains (0.3743 ± 0.03115), *p* = 0.0006. TfR1 expression at E19, P2, and P7 brains were unchanged between CHORIO and sham brains when compared at their respective gestational and postnatal ages, Figure 4C.

#### 2.3.1. CHORIO-Induced Changes in Brain HO-1/HO-2 and HO-1/TfR1 Ratios

**Figure 5:**
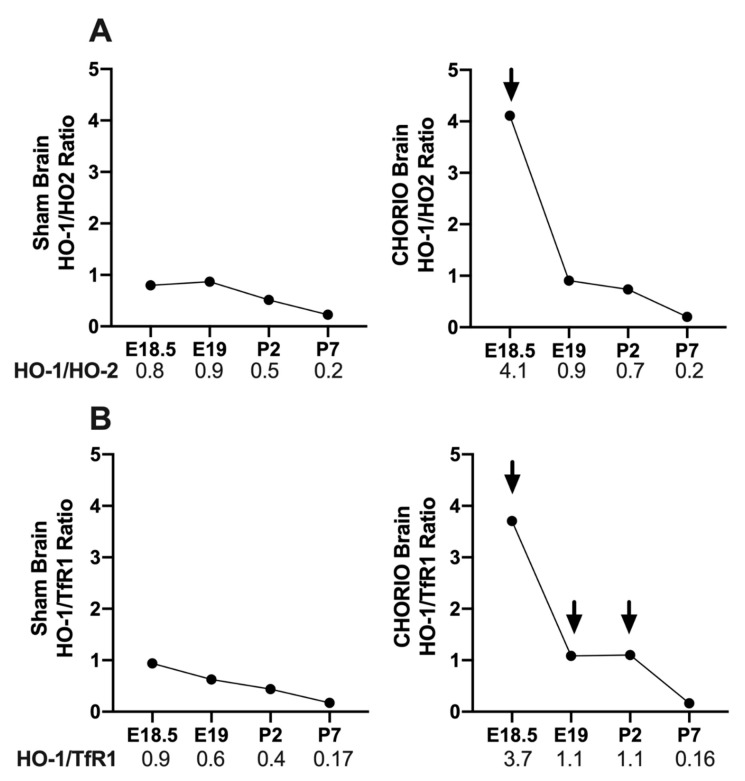
CHORIO-induced increase in HO-1/HO-2 and HO-1/TfR1 ratios in developing brain. Acute alteration of HO-1/HO-2 ratios in CHORIO brains compared with sham 6 h after initial insult (**A**). Initial acute alteration of HO-1/TfR1 ratios in CHORIO brains is sustained until P2, 7-days post initial insult in CHORIO brains compared with sham (**B**). Arrows indicate increased HO-1/HO-2 at E18.5 and HO-1/TfR1 at E18.5, E19 and P2 when compared with Sham.

HO-1 is an inducible stress enzyme and its upregulation may disturb cellular homeostasis resulting in cytotoxicity [14]. Therefore, we asked the question whether CHORIO disproportionally increased HO-1 relative to HO-2 and TfR1 in the brain. We demonstrated that at E18.5, 6 h after CHORIO, HO-1/HO-2 (4.1), Figure 5A, and HO-1/TfR1 ratios (3.7), Figure 5B, were altered in favor of excess HO-1, in fetal brains compared with sham brains (0.8 and 0.9, respectively). HO-1/HO-2 ratios at E19, P2, and at P7 were similar in CHORIO compared to sham. In contrast, HO-1/TfR1 ratios remained elevated at E19 (1.1) and at P2 (1.1), following the acute increase at 6 h in CHORIO brains compared with sham (0.6 and 0.4, respectively), Figure 5B. Thus, CHORIO resulted in a sustain alteration of HO-1/TfR1 ratios. Upregulation of HO-1 with a relative decrease in TfR1 during this critical CNS development period may be associated with CHORIO-induced brain injury.

#### 2.3.2. CHORIO-Induced Increase in Mononuclear Cells in Brain at P7

**Figure 6:**
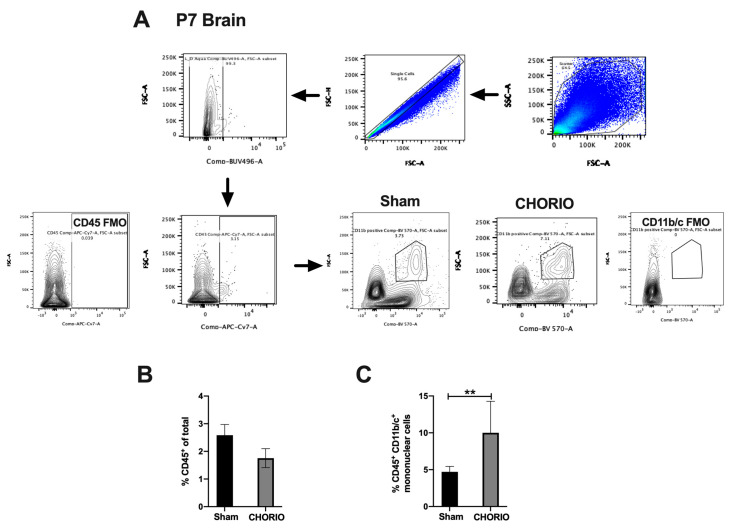
CHORIO-induced increase in brain mononuclear cells at P7. Single cells, live cells and CD45^+^ CD11b/c positive mononuclear cell population were identified by sequential gating. CD45 FMO and CD11b/c FMO controls were utilized to identify gating boundaries as shown in (**A**). No change in total CD45^+^ was detected between CHORIO and sham brains (*p* > 0.05, not normally distributed, Mann–Whitney) (**B**). Significant increase in the percentage of CD45 + CD11b/c^+^ mononuclear cells in CHORIO brains at P7 compared with sham (** *p* = 0.0098, normally distributed, Welch’s *t*-test, mean ± SEM), (**C**).

FIRS induced by CHORIO is a systemic multiorgan disease that results in neuroinflammation and brain injury in the neonate [6]. Thus, we examined whether CHORIO resulted in significant neuroinflammation at term equivalent age. Gating strategy is shown in Figure 6A. Total percentage of CD45^+^ leukocytes isolated from sham and CHORIO brains at P7 were similar (*p* > 0.05), Figure 6B. However, we detected a significant increase in the CD45^+^ CD11b/c^+^ mononuclear cells in CHORIO brains (10.0 ± 1.514, *n* = 8) at P7 compared with sham (4.71 ± 0.3649, *n* = 4, *p* = 0.0098), Figure 6C.

### 2.4. Peripheral Blood T Cell Composition at P21 after CHORIO

**Figure 7:**
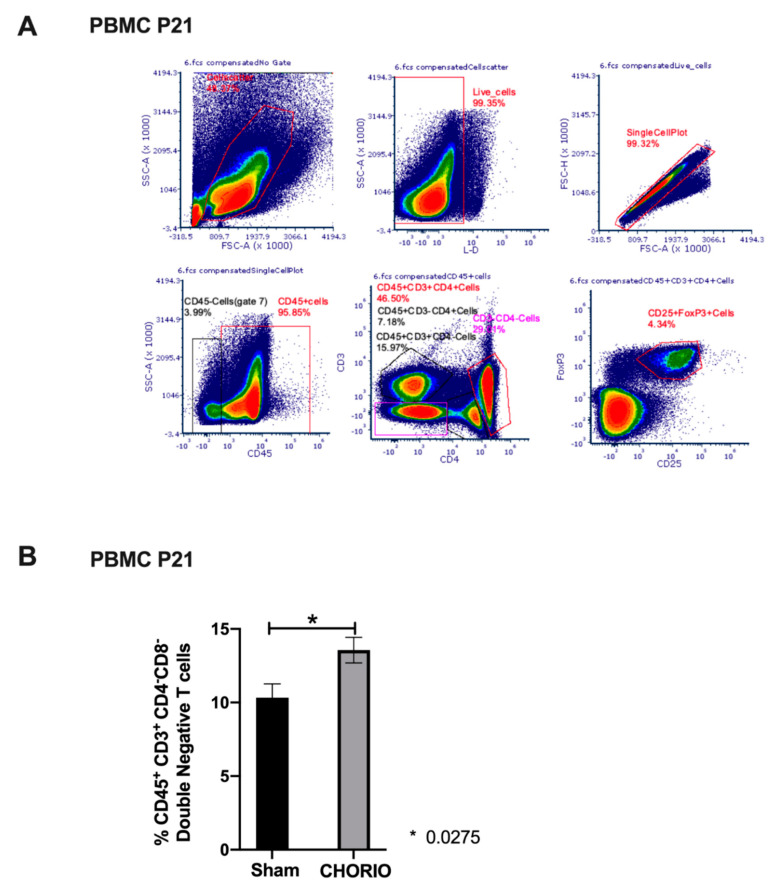
CHORIO induced increase in peripheral blood CD45^+^CD3^+^CD4^−^CD8^−^ T cells at P21. Representative gating strategy for PBMC analysis from a CHORIO pup at P21 (**A**). FMO controls were utilized for all fluorophores in the panel. CHORIO resulted in a significant increase in the percentage of CD45^+^CD3^+^CD4^−^CD8^−^ T cells from P21 pups compared with sham (* *p* = 0.0275, normally distributed, Welch’s *t*-test, mean ± SEM), (**B**).

Former preterm neonates who have CP have a persistent pro-inflammatory profile in peripheral blood at childhood [30]. Therefore, we set out to determine whether CHORIO in our model resulted in a pro-inflammatory adaptive immune programming at P21, at young adolescence. We measured adaptive immune responses in peripheral blood T cell subsets in sham and CHORIO pups at P21, a representative gating strategy is shown in Figure 7A. We did not detect significant alterations in percentages of gated peripheral blood T cell subsets, including CD45^+^CD3^+^ lymphocytes, CD3^+^CD4^+^ T helper (Th) cells, CD3^+^CD4^+^CD25^+^FoxP3^+^ T regulatory (Treg) cells, CD3^+^CD8^+^ T cells, and CD3^−^CD4^+^ immature T cells. However, %gated CD3^+^CD4^−^ T cells (double negative T cells) were significantly increased in CHORIO (13.56 ± 0.8722, *n* = 6) pups compared with sham (10.33 ± 0.09348, *n* = 8) at P21 (*p* = 0.0275), Figure 7B.

## 3. Discussions

Extremely premature neonates are born at a critical CNS developmental window [4]. Up to 40% of neonates who are born between 25–28 weeks of GA endure the highest risk of being exposed to CHORIO, developing CP and Neurodevelopmental Impairment (NDI) [4]. Studies in humans showed the association of early inflammation with later adverse neurodevelopmental and immune sequelae that expands well into school age [30,31]. Ultimately, complex interactions between an unfavorable intrauterine environment with inflammation/infection and genetic susceptibility or resilience can contribute to these sequelae. However, the exact mechanisms of how altered intrauterine environment in CHORIO leads to neuroimmune alterations and lifelong disability is unknown and still being investigated. Using an established model of CNS injury from prenatal chorioamnionitis [27] that replicates many features of Encephalopathy of Prematurity (EoP) [26,32], we demonstrate for the first time that CHORIO disrupts the normal ontogeny of HO pathway in the developing brain. Specifically, we show an upregulation of HO-1 and a sustained increase in HO-1/TfR1 ratios through P2, approximately 32 weeks GA in humans, after CHORIO insult at E18 (human equivalence of 24–25 weeks GA) compared to controls. This is associated with sustained neuro- and peripheral-inflammation as evidenced by an increase in mononuclear cells in CNS at P7, term equivalent age in humans and increase in double negative T cells in peripheral circulation at P21 (young adolescence). This CHORIO model replicates the effects of a transient systemic placental perfusion defect and concomitant inflammation encountered at a GA-equivalent to 25 weeks in human pregnancy [24]. The acute white matter injury, chronic microglia activation, astrogliosis and ventriculomegaly, along with persistent white matter injury, and the chronic myelination defects with devastating functional deficiencies through adulthood in this rat CHORIO model affirm the full spectrum of EoP observed in survivors of extreme prematurity [26].

We found an increase in the ratio of HO-1 to TfR1. Disturbance of HO-1/TfR1 ratios with a relative decrease in TfR1 is important because TfR1 is present in developing oligodendrocytes and absent from mature oligodendrocytes [33,34]. Persistent white matter injury is a hallmark of preterm brain injury from chorioamnionitis [32]. Furthermore, TfR1 is important in iron regulation which is essential for developing brain [35]. When taken together, the sustained increase in HO-1/TfR1 ratio, with a relative deficiency of TfR1 following CHORIO at the critical CNS developmental window of E18-P2 may highlight an important link between increased oxidative microenvironment and inflammation. The sustained postnatal upregulation of HO-1, persistent dysregulation of HO-1/TfR1 homeostasis and increased mononuclear cells in brains of CHORIO exposed pups highlight the importance of HO-1 pathway in brain-immune and neuroimmune responses. Our results suggest that homeostasis in HO-1 pathway may have a regulatory role in normal brain development. Hence, the role of inflammation-induced alterations in HO-1 pathway homeostasis that we demonstrate in neonatal WMI requires further investigation.

In the absence of inflammation at baseline, HO-1 is very low at birth, peaks at P7, followed by a 2–3-fold decrease at P21 with its lowest levels observed in adult brain [36,37]. In our current study, sham brains show an age dependent increase in HO-1 from E18.5 to P2, consistent with the literature. This difference in peak HO-1 expression in brain is attributable to technical differences between studies; we studied HO-1 expression by qPCR/mRNA compared with WB/IHC in the literature [36,37]. Furthermore, the earliest timepoint in Bergeron et al. study was P7 and all of the pups were naïve whereas in our study we studied much earlier timepoints with sham and CHORIO pups. While the HO-1 biology is complex and that are many transcriptional and post-transcriptional factors to consider with development and injury, mRNA expression typically precedes protein upregulation. Additionally, at baseline, in the developing rat brain, other researchers have shown that HO-2 gradually starts to increase postnatally approximately around P7 and continues to increase through lifetime into adulthood [38]. It is true that at any point in time brain HO-2 levels are higher than HO-1. Thus, this is a very delicate balance to maintain. It is noteworthy that Bergeron et al. study demonstrated regionality of brain HO-1 regulation in the absence of inflammation such that; in P7 developing brain HO-1 was highest by immunohistochemistry in white matter, cerebral cortex, hippocampus, thalamus, hypothalamus, and endothelial cells, while adult distribution of HO-1 immunoreactivity appeared at P21 [37]. We detected major disturbances in HO-1 and HO-1/TfR1 ratios at P2 in CHORIO brains.

Ontogeny of TfR1 in developing rat brain in association to HO pathway and CHORIO has not been previously studied. We demonstrated an acute increase in brain HO-1/TfR1 after CHORIO, which remained elevated at E19 and at P2, 7 days after initial insult. This suggests a disproportionately high HO-1 that is not balanced by TfR1, could lead to a locally altered iron metabolism and an altered oxidoreductive environment in CHORIO brains extending to P2, and not normalizing until P7 after initial insult. Needless to say, E18-P2 marks a critical CNS developmental window that is central to development, maturation, and migration of oligodendrocytes and neurons despite myelination will expand into P35 [39]. In fact, after hypoxic ischemic cerebral injury in P10 mice intranasally administered human apotransferrin resulted in recovery of WMI via facilitating OPC proliferation and inhibiting apoptotic cell death [40,41]. These findings, despite in a HIE model, underline the importance of our findings in the HO-1/TfR1 homeostasis and suggest that relative decrease in TfR1 can have a putative role in WMI and CP in CHORIO.

In our current study, we demonstrate that approximately 2-weeks after the original insult at P7, brains from CHORIO pups show a significant increase in brain mononuclear cell population compared to sham. In our future studies, we aim to investigate further, whether the sustained inflammation in CHORIO brains associated with sustained upregulation of HO-1, suggesting contribution of altered HO-1 pathway and relative decrease in TfR1 would lead to a putative increase in brain mononuclear cells and inhibition and stimulation experiments targeting HO-1/TfR1 pathway to show causality. We aim to show in our future studies the polarization status of these mononuclear cells, intracellular HO-1 expressions, TfR1 expressions in these cells and determine the distinction of microglia and brain infiltrating mononuclear cells. It is important to note that P7 is a critical timepoint in rodents as first postnatal week is the beginning of myelination [42]. Therefore, alteration of HO homeostasis in CHORIO brains can be a predecessor to WMI in EoP and CP. Furthermore, our results with significant increase in mononuclear cells at P7 CHORIO brains are in alignment with our previous immune findings in this model. Previously, we showed that at P7, CD45^+^CD11b/c^+^ peripheral blood mononuclear cells (PBMC’s) are significantly increased in CHORIO compared with sham [28]. Hence, here we show evidence that neuroinflammation correlates with peripheral inflammation in CHORIO, which could have implications for identifying and utilizing peripheral blood biomarkers for determining the risk of EoP and CP in a premature neonate for allow for targeted therapies.

Our results showing no change in peripheral blood conventional T cell subsets (CD45^+^CD3^+^ lymphocytes, CD3^+^CD4^+^ T helper (Th) cells, CD3^+^CD4^+^CD25^+^FoxP3^+^ T regulatory (Treg) cells, CD3^+^CD8^+^ T cells) are in alignment with human CHORIO offspring literature in our current study. For instance, cord blood from CHORIO exposed neonates have similar percentages and phenotype of Tregs compared to no CHORIO [43]. Importantly, at P21, we demonstrated an increase in %gated CD3^+^CD4^−^ cells in our current study. This population was also negative for CD8. Double negative (CD3^+^CD4^−^CD8^−^) T cells are comprised of two distinct populations; a pathogenic proinflammatory effector and a regulatory anti-inflammatory subset [44]. These cells are identified from both rodents and humans and are shown to be associated with various chronic inflammatory diseases [44]. Furthermore, a recent study by Meng et al. showed increased CD3^+^CD4^−^CD8^−^ T cells from peripheral blood and brains, promoted neuroinflammation via TNF-α dependent pathway, in a mice arterial occlusion ischemic stroke model [45]. In that study, CD3^+^CD4^−^CD8^−^ T cells co-localized with activated microglia and contributed to activation of microglia by TNF-α secretion [45]. Previously, we showed that the cytokine storm at E19 with significant elevations in pro-inflammatory cytokines TNF-α, IL-β, IL-6, CXCL-1, and anti-inflammatory cytokine IL-10, 24 h after CHORIO [24]. This persisted in offspring serum at P0 with sustained elevations in pro-inflammatory cytokines TNF-α and IL-6 and anti-inflammatory IL-10 and INF-γ, a pro-inflammatory cytokine with anti-inflammatory properties, 5-days after exposure [24]. Importantly, in this model, fetal CNS CXCL1 is significantly elevated at E19 [3]. We will need to conduct further studies for the functional characterization and the origins of these peripheral double negative T cells and to determine the contribution of double negative T cell subsets to the sustained peripheral hyperinflammatory response and neuroinflammation in cerebral palsy that we observe in our CHORIO model.

Our study has some limitations. We did not study the HO-1 pathway in later timepoints or determine regional or cell specific differences in the brain for HO-1, HO-2, and TfR1 expressions. We focused on earlier timepoints after CHORIO along with brain mononuclear cells at P7 to demonstrate the immediate effects of CHORIO in early neonatal period. We previously published PBMC secretory profiles at P7 and P21 and mononuclear cells in peripheral blood at P7. By studying the adaptive immune system at P21 we provide further evidence in our model that CHORIO results in a persistent immune dysregulation. We did not study functional status, polarization and distribution of CNS microglia, mononuclear cells in the context of their individual HO-1/TfR1 expressions. However, these are the focus of our future studies. Likewise, it will be important to show protein expression of HO-1/TfR1 in the brain. Additionally, other immune organ level adaptive immune system alterations in CHORIO remains to be determined in our model.

## 4. Conclusions

Collectively, our results highlight the importance of studying HO-1 pathway homeostasis and alterations in the developing brain and neuroinflammation associated with CHORIO. Our data support the hypothesis that, CHORIO disrupts the normal HO-1 and TfR1 ontogeny in utero and in the early perinatal period. Because discrete regulation of HO-1, HO-2, and TfR1 is important for homeostatic ontogeny, oxidoreductive balance, iron metabolism and immune regulation, these may be fundamental to neurodevelopment, neuroinflammation and sustained peripheral immune reactivity. More studies are needed to define the consequences of CHORIO on altered HO-1 signaling in the developing brain, specifically whether our observed findings are causal to development of cerebral palsy.

## 5. Materials and Methods

### 5.1. Institutional Animal Care and Use Committee (IACUC) Approval

All experiments and procedures were approved by the Johns Hopkins University School of Medicine IACUC.

### 5.2. Induction of Chorioamnionitis

Consistent with prior publications, we used a well-established model of CHORIO in Rats [24,26,27,28,46,47]. Pregnant Sprague–Dawley rats were obtained from Charles River Laboratories (Wilmington, MA, USA). On embryonic day 18 (E18), a laparotomy was performed under isoflurane anesthesia (4% for induction, 1% maintenance). CHORIO was induced by transient uterine artery occlusion (60 min), followed by intra-amniotic injections of lipopolysaccharide (LPS, *Escherichia coli*, serotype O111:B4, Sigma; 4-μg/sac). Sham control animals received laparotomy and externalization of the uterus, without uterine artery occlusion or LPS injection. Anesthesia times were equivalent between sham and CHORIO surgeries. After closing the laparotomy and providing appropriate pain control, CHORIO and sham pregnant dams recovered in their designated cages. Dams were closely monitored for any sign of distress or illness and delivered their litters at E22. Based on our previous studies, and in our experience, percentage of survival in Sham pups are 90% and CHORIO pups are 60% at P0 without any further mortality in the neonatal period [26]. All offspring were housed with their birth mothers. Consistent with NIH recommendations, we used both sexes in all experiments.

### 5.3. Tissue Collection

Brains were collected from CHORIO and sham pups (*n* = 3–10) at E18.5, E19, P2, and P7. Sham brains for HO-1 (*n* = 3, 5, 10, 7), HO-2 (*n* = 6, 8, 7, 7), and TfR1 (*n* = 7, 8, 6, 7) at E18.5, E19, P2, and P7 respective postnatal ages and CHORIO brains for HO-1 (*n* = 8, 4, 3, 7), HO-2 (*n* = 6, 3, 5, 7) and TfR1 (*n* = 7, 6, 6, 7) were used at E18.5, E19, P2, and P7 respective postnatal ages. Placentas were collected from CHORIO (*n* = 3–4) and sham (*n* = 3) dams at E19. Tissues were rapidly flash frozen and stored at −80 °C until used for experiments. Blood was collected at P21 and used fresh (Sham *n* = 8, CHORIO *n* = 6).

### 5.4. Extraction of RNA and Quantitative Real Time Polymerase Chain Reaction (RT-qPCR)

RNA was isolated from E18.5, E19, P2, and P7 CHORIO and sham brains, as well as from E19 placenta. Briefly, after homogenizing the tissue, total RNA was extracted from fetal and postnatal brains using direct-zol RNA Miniprep quick protocol (Zymo research, catalog no: R2070-71-72-73, Irvine, CA, USA) and from placentas using quick RNA Miniprep-quick protocol (Zymo research, catalog no: R1054, R1055, Irvine, CA, USA). We measured RNA concentration and purity by NanoDrop and proceeded with cDNA reaction using Bio-Rad 5x iSCRIPT reverse transcription SuperMix for RT-PCR on a MiniAmp thermal cycler (Thermo Fisher, Waltham, MA, USA). 900 ng of RNA was converted to cDNA for brains and 300 ng for placenta. After which, RT-PCR was performed using SYBR green on a Quant Studio-3 (Thermo Fisher, Waltham, MA, USA). CT values were compared to their pooled reference naïve tissues at E19 and normalized to 18S endogenous control as previously published [26]. We use 18S endogenous control as 18S expression is stable over developmental periods in our model [26]. We verified the primer sequences using the Basic Local Alignment Search Tool (BLAST) for Nucleotides on National Center for Biotechnology Information (NCBI) website. Primer sequences for HO-1, HO-2, TfR1, and 18S are listed in Table 1. All samples were run in triplicates. CT values > 0.25 standard deviations were excluded from analysis.

#### 5.4.1. Isolation of Single Cells (Brain)

Single cell suspensions from brains were yielded using Neural Tissue Dissociation Kit (*p*) from Miltenyi Biotec (Auburn, CA, USA) following the manufacturer’s protocol. Briefly, pups were euthanized by decapitation at P7 and blood was collected. Brains were immediately placed on ice cold Hank’s Balanced Salt Solution (HBSS) (without Ca^+^ and Mg^+^) and weighed. For each sample, 200–400 mg of brain tissue was utilized and samples were not pooled. After removing cerebellum and frontal lobes via sterile blade, right and left hemispheres from individual brains were placed in a pre-heated (37 °C) proprietary enzyme mix (Miltenyi, Neural Tissue Dissociation Kit) and serial mechanical dissociation, enzymatic digestion steps were completed on Miltenyi Gentle MACS Dissociator and MACS Mix Tube Rotator, respectively. Subsequently, cells were filtered through a BD Falcon 70-μm cell strainer, washed with HBSS, and resuspended in PBS. Next, isolated brain cells in PBS were carefully layered onto

Ficoll Paque Plus (GE Healthcare, Chicago, IL, USA), brain mononuclear cells were separated by density gradient centrifugation and collected for further flow cytometry applications.

#### 5.4.2. Isolation of Single Cells (Blood)

Blood was collected by terminal intracardiac puncture at P21 from sham and CHORIO pups in heparinized syringes, immediately mixed with PBS, and placed on ice. Peripheral Blood Mononuclear Cell Layer (PBMC’s) was separated by Ficoll Paque Plus (GE Healthcare, Chicago, IL, USA) density gradient technique, following the manufacturer’s recommendation for further flow cytometry steps.

### 5.5. Multiparameter Flow Cytometry

The following antibodies were purchased from BD Bioscience (San Jose, CA, USA) for mononuclear cell panel, anti-CD45 APC Cy7, anti-CD45 PerCP-Cy5.5, anti-CD11b/c BV605, Purified mouse anti-rat CD32 Fc block and from BioLegend (San Diego, CA, USA), BD Bioscience (San Jose, CA, USA) or Novus Biologicals (Littleton, CO) for T cell panel, anti-CD45-PerCP-Cy5.5, anti-CD3 APC, anti-CD4 APC-Cy7, anti-CD8 Alexa Fluor 594, anti-CD25 BV421, anti-FoxP3 FITC and Live/Dead fixable Aqua Dead Cell Stain Kit, for 405 nm from Thermo Fisher (Waltham, MA, USA). Cells were counted on a Countess^TM^ II FL Automated Cell Counter (Thermo Fisher, Waltham, MA, USA). Single cell brain mononuclear cell layer was incubated with viability dye in PBS for 30 min on ice, followed by Fc block with anti-CD32 and antibody staining in MACS buffer. Peripheral blood mononuclear cell layer was first incubated with viability dye in ice cold PBS for 30 min, followed by antibody staining in MACS buffer. OneComp eBeads were used for compensation. Unstained similarly treated brain cells and PBMC were used to detect autofluorescence and fluorescent minus one (FMO) controls were utilized for proper gating.

Brain multiparameter flow cytometry was performed on a LSRII (BD Bioscience, San Jose, CA, USA) and results were analyzed by FlowJo 10.6.1 (FLowJo Portal, Becton-Dickinson, Ashland, OR, USA). PBMCs multiparameter flow cytometry was performed on a Cytek Aurora and analyzed on FCS Express Plus 7 (De Novo Software, Pasadena, CA, USA).

### 5.6. Identification of Immune Cells and Phenotyping

We excluded debris and doublets and identified single cells by sequential gating on forward scatter height versus forward scatter area. After gating on live cells, for the brain panel, we sequentially gated on CD45^+^ cells and CD11b/c^+^ cells. We identified CD45^+^ leukocytes and CD45^+^CD11b/c^+^ mononuclear cell populations in Sham and CHORIO brains at P7, 2 weeks after exposure to CHORIO. For the PBMC panel, debris and doublets were similarly excluded, live and single cells and CD45^+^ leukocytes were identified. Then by sequential gating we identified CD3^+^CD4^+^, CD3^+^CD4^+^CD25^+^FoxP3^+^, CD3^+^CD4^−^, CD3^−^CD4^+^, CD3^+^CD8^+^ T cell subsets and FoxP3 median fluorescent intensity (MFI).

### 5.7. Statistical Analysis

Data were analyzed using GraphPad Prism Software Version 8.3.0 using a Mann–Whitney, Kruskal–Wallis with Dunn’s multiple comparisons test for not normally distributed data, Welch’s *t*-test, 2-tailed for normally distributed data or two-way ANOVA. Data are expressed as median or mean ± standard error of the mean (SEM). Based on our previous experience with the CHORIO model, and assuming a power of 0.8 and an alpha of 0.05 for multiple group comparisons, 3–8 samples per experiment is sufficient for power and statistical significance [26,46]. Results are deemed statistically significant when *p* < 0.05.

## Figures and Tables

**Table 1:** Primers used for RT-PCR.

Target	Primer Sequence
HO-1 (Inducible HO)	(F)5′CCTTCCCGAACATCGACAGCC3′ (R)5′GCAGCTCCTCAAACAGCTCAA3′
HO-2 (Constitutive HO)	(F)5′GGAGGGGGTAGATGAGTCAGA3′ (R)5′TCGGTCATGTGCTTCCTTGGT3′
TfR1 (Transferrin R)	(F)5′AGTTGAACAAAGTGGCACGAGCAG3′ (R)5′AGCAGTTGGCTGTTGTACCTCTCA3′
18 S	(F)5′TCCCTAGTGATCCCCGAGAAGT3′ (R)5′CCCTTAATGGCAGTGATAGCGA3′

## Data Availability

All data supporting the reported results are included in the manuscript and the Supplementary Material.

## Supplementary Materials

The following are available online at https://www.mdpi.com/article/10.3390/ijms22115773/s1.
