# Supplementary material for: Chorioamnionitis Precipitates Perinatal Alterations of Heme-Oxygenase-1 (HO-1) Homeostasis in the Developing Rat Brain
Source: Int J Mol Sci. 2021 May 28;22(11):5773. doi: 10.3390/ijms22115773 (PMC8198804; doi:10.3390/ijms22115773)
Supplement: Supplementary file 1 [file ijms-22-05773-s001.zip › SupplementalFigureLegend_April17_2021.pdf]

Supplemental Figure 1. Determination of intraamniotic LPS treatment effect. 2-way ANOVA results showing significant treatment effect of CHORIO on brain HO-1 expression (FIG **S1A**, $p=0.0092$) but not on HO-2 or TfR1. Whereas, HO-2 (**FIG S1B**) and TfR1 (**FIG S1C**) were regulated significantly by postnatal age but not by treatment effect. p significant when <0.05, 2-way ANOVA, Mean±SEM.
