# Supplementary figures and images for: Chorioamnionitis Precipitates Perinatal Alterations of Heme-Oxygenase-1 (HO-1) Homeostasis in the Developing Rat Brain
Source: Int J Mol Sci. 2021 May 28;22(11):5773. doi: 10.3390/ijms22115773 (PMC8198804; doi:10.3390/ijms22115773)

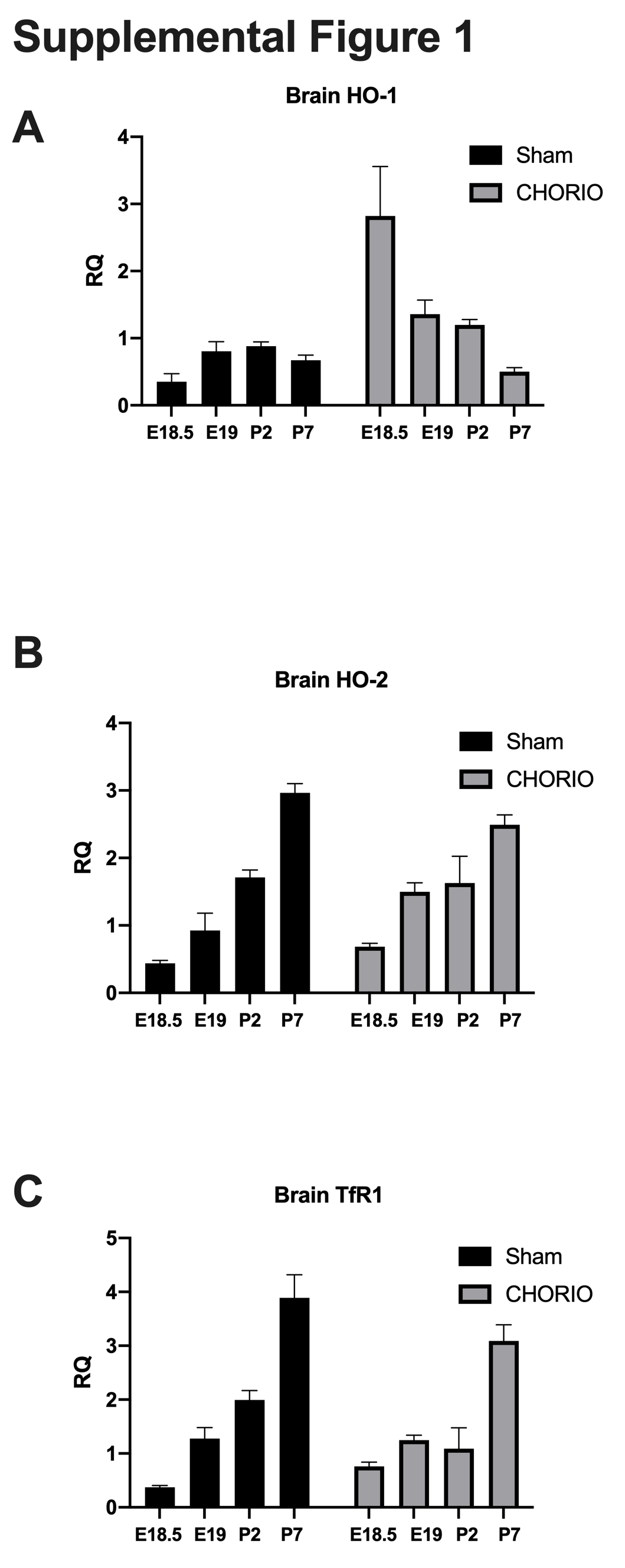

Supplement: Supplementary file 1 [file ijms-22-05773-s001.zip › Supplemental Figure 1_2021_Jan13_Two-way ANOVA Brain Sham CHORIO E18+6 to P7 (1).tiff]
